# Seroreversion of IgG anti‐HEV in HIV cirrhotic patients: A long‐term multi‐sampling longitudinal study

**DOI:** 10.1111/tbed.14486

**Published:** 2022-03-03

**Authors:** Pedro López‐López, Mario Frias, Angela Camacho, Isabel Machuca, Javier Caballero‐Gómez, María A. Risalde, Ignacio García‐Bocanegra, Ignacio Pérez‐Valero, Jose C. Gomez‐Villamandos, Antonio Rivero‐Juárez, Antonio Rivero

**Affiliations:** ^1^ Infectious Diseases Unit and Clinical Virology and Zoonoses Unit, Maimonides Institute for Biomedical Research, Reina Sofia Hospital University of Cordoba Cordoba Spain; ^2^ CIBERINFEC; ^3^ Animal Health and Zoonoses Research Group (GISAZ), Animal Health Department University of Cordoba Cordoba Spain; ^4^ Animal Health and Zoonoses Research Group (GISAZ), Animal Pathology and Toxicology Department University of Cordoba Cordoba Spain

**Keywords:** antibodies, cirrhotic, HCV, HEV, HIV, seroreversion

## Abstract

The aim of our study was to evaluate HEV antibody kinetics in HIV/HCV‐coinfected patients with cirrhosis. A longitudinal retrospective study was designed. Patients were followed up every 6 months; anti‐HEV IgG and IgM antibodies levels and HEV‐RNA by qPCR were analysed. The prevalence and incidence of every HEV infection marker were calculated. The kinetics of anti‐HEV IgG and IgM during the follow‐up were evaluated. Seventy‐five patients comprised the study population. The seroprevalence observed was 17.3%. None showed IgM antibodies or HEV‐RNA at baseline. None showed detectable HEV viral load during the study period. After a median follow‐up of 5.1 years, two of 62 seronegative patients (3.2%) seroconverted to IgG antibody. The incidence for IgM was 2.7%. Of the 13 patients with IgG seropositivity at baseline, five (38.5%) seroreverted. Meanwhile, of the two patients who exhibited IgM positivity during the study, one (50%) showed intermittent positivity. We found that HEV seropositivity is common in HIV/HCV‐coinfected cirrhotic patients. A remarkable rate of IgG seroreversions and IgM intermittence was found, limiting the use of antibodies for the diagnosis of HEV infection in this population.

## 1 INTRODUCTION

Hepatitis E virus (HEV) is the most common cause of acute hepatitis around the world and, consequently, is a major global health issue (Kamar et al., 2014). In most cases, HEV infections are usually subclinical and self‐limiting (Hoofnagle et al., 2012). However, HEV infection could have a worse prognosis in several groups of patients, such as immunosuppressed patients (Rivero‐Juarez et al., 2019), pregnant women (Karna et al., 2020) or patients with underlying chronic liver disease (Kamar et al., 2012).

For the diagnosis of HEV infection, both serological and molecular markers are employed (Lu et al., 2021). The most sensitive method is the detection of HEV RNA, which allows the diagnosis of both acute and chronic infections. Nevertheless, its use requires more complex and expensive methods than HEV antibody detection. For this reason, clinical guidelines recommend the detection of anti‐HEV IgM as a screening approach for the diagnosis of acute HEV infection (European Association for the Study of the Liver, 2018; Rivero‐Juárez et al., 2020). In contrast, the presence of IgG antibodies is indicative of past infection, thus is not of diagnostic value but is useful for epidemiological studies. Therefore, anti‐HEV IgG antibody levels could be used to identify individuals who could be protected against HEV in the long term (Su et al., 2017; Zhang et al., 2014). However, several studies highlight that the clinical value of antibody determination (IgG and IgM) could limit the diagnostic value and, consequently, jeopardize the management of patients with HEV. First, it has been reported that the loss of IgG antibodies (seroreversions) in immunosuppressed patients might imply the risk of reinfection in these patients (Abravanel et al., 2014). Second, it has been communicated that IgM could persist for a long time; thus, its use for the diagnosis of acute infection could be limited. Consequently, studies evaluating the kinetics of HEV antibodies over time are needed to optimize the management of HEV infection in high‐sensitivity populations. For these reasons, we designed a study to evaluate the anti‐HEV antibody kinetics in a cohort of HIV‐infected patients with cirrhosis.

## 2 MATERIALS AND METHODS

### 2.1 Study design and patients

This was a longitudinal retrospective study including HIV cirrhotic patients in follow‐up at the Hospital Universitario Reina Sofia de Cordoba (Spain) between January 2012 and October 2020. Inclusion criteria in the cohort were as follows: (i) diagnosis of liver cirrhosis by transient liver elastography (Vergara et al., 2007) or F4 METAVIR fibrosis score by histological examination, and (ii) no history of liver decompensation prior to inclusion in the cohort. Patients were followed up every 6 months collecting a blood sample for the analysis.

### 2.2 Variable collection and definition of HEV infection

In all the samples, anti‐HEV IgG and IgM antibodies and HEV RNA were evaluated. HEV infection was defined as detectable HEV‐RNA and/or positivity to IgM, independent of the IgG result. Regarding HEV antibodies, four categories were defined to classify the serostatus of the patients: (i) HEV seropositivity, defined as positivity to IgG and/or IgM antibodies at baseline, (ii) HEV seroconversion, defined as positivity to anti‐HEV IgG and/or IgM antibodies during the follow‐up in patients with negative markers in the previous visit, (iii) IgG seroreversion, defined as undetectable anti‐HEV IgG antibody in patients with a previous positivity, and (iv) persistence of anti‐HEV IgM antibody, defined as the positivity of anti‐HEV IgM antibodies for more than 6 months (Goel & Aggarwal, 2020).

### 2.3 Molecular and serological determination for HEV

Serum was obtained by centrifugation for 10 min at 400 × *g* and stored at −80°C until required for analysis. Samples were tested for anti‐HEV IgG and anti‐HEV IgM antibodies by commercial ELISA (recomWell HEV IgG/IgM®; Mikrogen Diagnostik, Neuried, Germany) using an automated procedure (Automatic ELISA workstation DS2®, Dynex Technologies). The analyses were carried out in accordance with the instructions provided by the manufacturer using a cut‐off value ≥ 24 U/ml for positive samples. Antibodies quantitative value are presented in U/ml, not related with the WHO international standard. The specimens with a value‐to‐cut‐off ratio between 20 and 24 U/ml were considered borderline. Confirmatory testing was performed using immunoblotting (recomLine HEV IgG/IgM®; Mikrogen Diagnostik, Neuried, Germany), following a manual procedure according to the manufacturer's instructions for all positive samples. RNA was extracted from 400 μl of serum using the commercial QIAamp Mini Elute Virus Spin Kit (QIAgen, Hilden, Germany) by an automated procedure (QIAcube. QIAgen, Hilden, Germany). The purified RNA was eluted in a volume of 50 μl. RT q‐PCR for HEV was performed using the QIAgen One‐Step PCR Kit (QIAgen, Hilden, Germany), following an in‐house protocol described previously by our group (Frías et al., 2021).

### 2.4 Ethics statement

This study was designed and conducted in accordance with the Declaration of Helsinki. The Ethics and Clinical Trials Committee (CEIC) of Córdoba approved the study protocol, obtaining the informed consent of each patient. The SSPA Biobank has coordinated the collection, processing, handling and assignment of the biological samples used in this study in accordance with the standard procedures established for this purpose.

### 2.5 Statistical analysis

Continuous variables were expressed as the median and interquartile range (IQR) (Q1–Q3). Student's *t* test, the Welch test or the Mann–Whitney *U*‐test was used to compare two independent variables. Categorical variables were expressed as the number of cases (percentage) and compared using the *χ* ^2^ test or the Fisher's exact test. The prevalence of HEV infection was calculated as the number of patients who fulfilled the primary outcome variable divided by the total number of patients included in the study at baseline. Incidence was calculated by the ratio of new events (seroconversion) and the number of seronegative patients at the beginning of the study. The incidence rate was calculated as the number of new events (seroconversions) divided by the accumulated follow‐up time per 1000 patient‐years. The proportion of patients with IgG seroreversion was also calculated. For all of them, a two‐sided 95% confidence interval (95% CI) was calculated based on exact binomial distributions. Analyses were carried out using the SPSS statistical software package version 18.0 (IBM Corporation, Somers, NY, USA).

## 3 RESULTS

### 3.1 Study population

Seventy‐five patients were included in the study. Of these, 67 (89.3%) were males and eight (10.6%) were females, with a median age of 53 years (IQR: 49–56 years). In Table 1, we show the baseline characteristics of the population. Seventy‐four patients (98.6%) were treated for HCV during the study, and 67 (90.5%) of them achieved sustained virological response (SVR). All patients were under antiretroviral therapy, all with undetectable HIV viral load. No patient received any blood transfusion or intravenous immunoglobulin therapy during the study.

**TABLE 1:** Description of the baseline characteristics of the study population

Patients	*n* = 75
Sex (male) (%)	67 (89.3%)
Age (years), median (Q1–Q3)	53 (49–56)
Parenteral drug user (%)	68 (90.6%)
Follow‐up (years), median (Q1–Q3)	5.1 (3.5–6.4)
ART (yes) (%)	75 (100.0%)
HIV (≤50 copies/ml) (%)	75 (100.0%)
HBsAg (positive) (%)	2 (2.6%)
Alcohol (uptake > 50 g/day) (%)	7 (9.3%)
AST (U/L), median (Q1–Q3)	51 (31.5–96)
ALT (U/L), median (Q1–Q3)	50 (27–99)
GGT (U/L), median (Q1–Q3)	108 (56–200)
Total bilirubin (mg/dl), (Q1–Q3)	1.1 (0.7–2)
Glucose (mg/dl), median (Q1–Q3)	93.5 (87–106.7)
Albumin (g/dl), median (Q1–Q3)	4 (3.7–4.3)
CRP (mg/L), median (Q1–Q3)	1.1 (0.5–4)
Creatinine (mg/dl), median (Q1–Q3)	0.81 (0.74–0.94)
Sodium (mEq/L), median (Q1–Q3)	139 (138–140)
Prothrombin (%), median (Q1–Q3)	86 (72.4–97.2)
Blood platelets (10^3^/μl), median (Q1–Q3)	109 (73–171)
CD4+ (cell/μl) median (Q1–Q3)	368.5 (152–479.2)
INR, median (Q1–Q3)	1.1 (1–1.2)
Child PT, A	35 (46.7%)
MELD, median (Q1–Q3)	9 (7–11)

Abbreviations: ART, antiretroviral therapy; HIV, human immunodeficiency virus; HBsAg, hepatitis B virus surface antigen; AST, aspartate aminotransferase; ALT, alanine aminotransferase; GGT, gamma‐glutamyl transferase; INR, international normalized ratio; MELD, model for end stage liver disease; Child PT, child turcotte pugh; Q, quartile; μL, microliter; mL, milliliter; dL, deciliter; L, litre; g, gram; mg, miligram; mEq, milliequivalent; CRP, C‐reactive protein.

### 3.2 Prevalence and incidence ratio of HEV seropositivity

**FIGURE 1:**
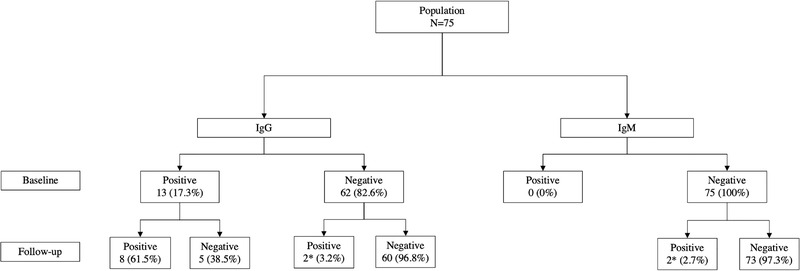
Seroprevalence and incidence of HEV infection, seroreversions of anti‐HEV IgG and seroconversions of anti‐HEV IgG/IgM at the end of the study. *One patient seroconverted to anti‐HEV IgG and IgM antibodies

The baseline prevalence of anti‐HEV IgG antibodies was 17.3% (13 out of 75 [95% CI: 10.3%–27.6%]). IgM antibodies or HEV‐RNA were detected in any of the patients. In Figure 1, we show the flow diagram of the patients.

After a median follow‐up of 5.1 years (IQR: 3.5–6.4 years), a total of 507 samples were analysed. The median samples analysed per individual was 7 (IQR: 5–8). During the study period, three patients experienced HEV antibodies seroconversion (Figure 1). One patient seroconverted for both anti‐HEV IgG and IgM antibodies, other for IgG and the other for IgM. Considering only those individuals who tested negative for antibodies at the baseline, it was determined a cumulative incidence of 3.2% (2 out of 62 [95% CI; 0.2%–11.7%]) and an incidence rate of 6.3 (0.66–36.96) cases per 1000 patient‐years. HEV‐RNA was not detected in any patient for each sample analysed during follow‐up in the study.

### 3.3 Seroreversion of anti‐HEV IgG

Seroreversions for anti‐HEV IgG antibody were evaluated by monitoring 13 patients who were positive at baseline. At the end of the follow‐up, 38.5% of the patients (5 out of 13 [95% CI; 17.6%–64.6%]) experienced loss of IgG antibodies (Figure 1).

Two of the five patients who experienced IgG antibody seroreversion showed intermittent IgG antibodies positivity during the follow‐up. Seroconversion timeline is shown in Table 2. The median time to seroreversion was 3.36 years (IQR: 2.19–4.1 years). The baseline median CD4+ count was 575.4 cells/μl (IQR: 347–863.5 cells/μl) for seroreverted patients, and no patient had a CD4+ count below 200 cells/μl at baseline or at the time of IgG antibody seroreversion. No differences were found between persistent IgG and seroreverted patients for age (55 [IQR: 52.25–61] vs. 54 [IQR: 52–57.5]; *p* = .491), sex (male 7 out of 8 [87.5%] vs. 5 out of 5 [100%]; *p* = .99) or CD4 + cell count (472 [IQR: 391–889] vs. 456 [IQR: 347–863]; *p* = .889). During the follow‐up, no patient who seroreverted for the anti‐HEV IgG antibody was positive for the IgM antibody and/or RT‐qPCR for any of the samples analysed at the different points after seroreversion. On the other hand, 40% (2 out of 5 [95% CI; 11.6%–77.1%]) of the patients presented intermittent positive IgG antibodies for at least one follow‐up point after seroreversion (Table 2). IgG and IgM antibodies values (U/ml) are shown in Table S1.

**TABLE 2:** Timeline of the dynamics of anti‐HEV IgG/IgM antibodies for those patients showing at least one positive result during the study

Patient ID	Visit 1 (IgG/IgM)	Visit 2 (IgG/IgM)	Visit 3 (IgG/IgM)	Visit 4 (IgG/IgM)	Visit 5 (IgG/IgM)	Visit 6 (IgG/IgM)	Visit 7 (IgG/IgM)	Visit 8 (IgG/IgM)	Visit 9 (IgG/IgM)	Visit 10 (IgG/IgM)	Interpretation
1	Neg/Neg	Neg/Neg	Neg/Neg	Neg/Neg	Neg/Neg	**Pos**/Neg					IgG seroconversion
2	Neg/Neg	Neg/Neg	Neg/Neg	Neg/Neg	Neg/Neg	**Pos/Pos**					IgG and IgM seroconversion
3	**Pos**/Neg	**Pos**/Neg	**Pos**/Neg	**Pos**/Neg	**Pos**/Neg	Neg/Neg	Neg/Neg				IgG seroreversion
4	**Pos**/Neg	**Pos**/Neg	**Pos**/Neg	Neg/Neg	Neg/Neg	Neg/Neg	Neg/Neg	Neg/Neg			IgG seroreversion
5	**Pos**/Neg	Neg/Neg	Neg/Neg	Neg/Neg	Neg/Neg	Neg/Neg	Neg/Neg	Neg/Neg			IgG seroreversion
6	**Pos**/Neg	Neg/Neg	**Pos**/Neg	Neg/Neg							IgG intermittent seroreversion
7	**Pos**/Neg	Neg/Neg	Neg/Neg	Neg/Neg	Neg/Neg	**Pos**/Neg					IgG intermittent seroreversion
8	**Pos**/Neg	**Pos**/Neg	**Pos/Pos**	**Pos**/Neg	**Pos/Pos**						IgG persistence/ IgM intermittence
9	Pos/Neg	Pos/Neg	Pos/Neg	Pos/Neg	Pos/Neg	Pos/Neg	Pos/Neg	Pos/Neg	Pos/Neg	Pos/Neg	IgG persistence
10	Pos/Neg	Pos/Neg	Pos/Neg	Pos/Neg	Pos/Neg	Pos/Neg	Pos/Neg	Pos/Neg	Pos/Neg	Pos/Neg	IgG persistence
11	Pos/Neg	Pos/Neg	Pos/Neg	Pos/Neg	Pos/Neg	Pos/Neg	Pos/Neg	Pos/Neg			IgG persistence
12	Pos/Neg	Pos/Neg	Pos/Neg	Pos/Neg	Pos/Neg						IgG persistence
13	Pos/Neg	Pos/Neg	Pos/Neg	Pos/Neg							IgG persistence
14	Pos/Neg	Pos/Neg	Pos/Neg	Pos/Neg							IgG persistence
15	Pos/Neg	Pos/Neg	Pos/Neg								IgG persistence

Abbreviations: ID, identification; IgG, immunoglobulin G; IgM, immunoglobulin M; Pos, positive; Neg, negative.

### 3.4 Persistence of anti‐HEV IgM antibodies

Two patients (2.7%) presented seroconversion to IgM antibodies during the study (Figure 1). One of them seroconverted to IgG and IgM antibody in the last visit of the study (Table 2). At baseline, this patient presented 216 cells/μl, and during the study, the median was 189.5 cells/μl (IQR: 146–230.5 cells/μl). At the time of seroreversion, the patient showed CD4 titres above 200 cells/μl (Table S2). The other patient seroconverted to both IgG/IgM antibodies. This patient exhibited HEV IgM antibody persistence for more than 6 months, showing intermittent IgM antibody positivity in two determinations (Table 2), remaining positive for IgG antibody during the whole study period. At baseline, this patient presented 503 cells/μl, and during the study, the median was 501.5 cells/μl (IQR: 481.2–842.7 cells/μl). At no time did this patient have a CD4+ count below 200 cells/μl (Table S2).

### 3.5 Decompensation and death

A total of 15 patients (20%) presented liver decompensation during the study period. Eight (53.4%) developed ascites, three (20%) hepatic encephalopathy, two (13.3%) hypertensive gastrointestinal bleeding and two (13.3%) hepatocellular carcinoma. The median time up to decompensation was 1.49 years (IQR: 0.57–2.47 years). Seven (9.3%) patients died during the study: three (42.8%) died due to liver‐related events, two (28.6%) due to infections (multi‐lobular pneumonia and infection secondary to an intestinal perforation), one (14.3%) due to chronic renal failure and one (14.3%) due to non‐AIDS‐related comorbidities (mandibular squamous cell carcinoma). None of the decompensations presented by the patients was related to HEV infection.

No differences in liver decompensation (7.4% vs. 21.3%; *p* = .364) or mortality (14.3% vs. 8.2%; *p* = .608) were observed between the patients exhibiting IgG antibodies and those not at baseline. None of the seroconverted or seroreverted patients at the end of the study died.

## 4 DISCUSSION

In the natural course of HEV infection, after an approximate incubation period of 2 weeks, HEV‐RNA can be detected in blood up to approximately 3 weeks after the onset of symptoms (Kamar et al., 2014). Thereafter, an initial short‐lived IgM antibody response, persisting for 6 months, is followed by IgG antibodies (Kamar et al., 2014). Consequently, the detection of IgM indicates a recent infection (Goel & Aggarwal, 2020). However, several studies have found that anti‐HEV IgM antibodies may persist for more than 6 months (Myint et al., 2006; Norder et al., 2016; Riveiro‐Barciela et al., 2020). In a cohort of 25 patients with self‐limited acute HEV infection, it was found that 24%–56% (depending on the assay employed) of individuals showed positivity for anti‐HEV IgM antibodies at baseline and that IgM remained detectable after a median follow‐up of 34 months (Riveiro‐Barciela et al., 2020). Another study that retrospectively evaluated samples from 62 adults diagnosed with acute HEV during two outbreaks reported that 25% of individuals with positive IgM antibodies remained positive for IgM for at least 14 months (Myint et al., 2006). In addition, a study carried out in Sweden found one individual out of 27 who presented persistence of IgM antibodies but only for 7 months (Norder et al., 2016). Nevertheless, these studies present methodological differences from ours; we longitudinally analysed multiple samples during a long follow‐up, whereas the other studies analysed only baseline and final samples (Norder et al., 2016; Riveiro‐Barciela et al., 2020) or different nearby points during a short follow‐up (Myint et al., 2006). In our study, we do not find IgM persistence. Nevertheless, we found that IgM antibody positivity might fluctuate over time, showing intermittent positivity. This intermittence did not imply acute HEV infection because of the lack of symptoms or presence of HEV RNA. For these reasons, the only determination of IgM for the diagnosis of acute HEV may mean detecting a proportion of noninfected individuals. Therefore, it could be necessary to combine the use of serological and molecular markers to increase the sensitivity and specificity of the diagnosis of acute HEV infection (Rivero‐Juarez et al., 2021).

After the acute phase of the infection, anti‐HEV IgG antibodies are produced to persist for at least several years and confer immunity against HEV for a long time (Su et al., 2017; Zhang, 2014). Thus, IgG detection indicates previous exposure to HEV (Goel & Aggarwal, 2020). However, studies have shown that IgG seroreversions may occur in a proportion of patients (Faber et al., 2018), associated with immunosuppression (Kaba et al., 2011; Pineda et al., 2014) and with low baseline antibody titres (Servant‐Delmas et al., 2016). In our study, we found seroreversion for anti‐HEV IgG antibody in five out of 13 (38.5%) patients with a mean follow‐up of 3.36 years. This fact has important implications because the loss of acquired immunity could confer susceptibility to reinfection with HEV. However, we did not find recent infections in these patients during the study period, so we were unable to evaluate this point. Several factors associated with seroreversion have been identified. In one study, it was observed that a low CD4+ count (<200 cells/μl) was associated with seroreversions of the IgG antibody in HIV patients (Pineda et al., 2014). Another study conducted in a Swiss cohort including 735 HIV‐infected patients found that the prevalence of IgG antibody was lower in patients with low CD4+ counts (Kenfak‐Foguena et al., 2011). In our study, we did not find a relationship between CD4+ cell count and IgG antibody seroreversions, showing that all seroreverted patients had a high CD4+ cell count. However, it has been also shown that HIV‐infected individuals had a lower humoral response, independently of the CD4+ cells count (Abravanel et al., 2017). Thus, the seroreversion for IgG antibodies could be related with this, but could not evaluate this point in our study. Other factors affecting seroreversion should be evaluated.

On the other hand, we did not find HEV infection to be the main trigger of liver decompensation in cirrhotic HCV/HIV‐coinfected patients in our study. Of the 15 patients who decompensated in our study, none had liver injury associated with HEV infection. Similarly, studies carried out in cirrhotic patients in France or the United Kingdom found low liver decompensation due to HEV infection, 3.5% and 3.2%, respectively (Blasco‐Perrin et al., 2015; Haim‐Boukobza et al., 2015). In addition, a study conducted in the United States found an incidence of 4.5% anti‐HEV IgG antibodies in decompensated patients (Samala et al., 2016). These differences observed between Asia and America and Europe are not related to the prevalence and incidence of HEV infection in this population, because studies carried out in patients with chronic hepatitis C in the United States or with chronic liver diseases in Spain showed prevalences comparable to our study (Samala et al., 2016; Vázquez‐Morón et al., 2019). Likewise, the incidence rates reported in the United Kingdom (2/1000 patient‐years) and the United States (7/1000 patient‐years) were similar to those observed in our cohort (Khuroo et al., 2016). Therefore, in Europe and America, exposure to HEV among patients with underlying chronic liver disease seems to be common. For this reason, although HEV infection is frequent in patients with underlying chronic liver disease in countries where genotype 3 is the main cause of infection, this seems not to be a leading cause of liver decompensation or death in this population.

Several limitations should be noted. The main limitation of our study is the relatively low number of patients, which could be insufficient to identify any HEV infection as a risk factor for liver decompensation and death. However, the longitudinal nature of our study together with the multisample analysis allows us to observe the dynamics of anti‐HEV IgG/IgM antibodies of each individual at different points as well as assess their association with the possible outcomes. Furthermore, although the association of HEV with decompensation in cirrhosis has been found to be independent of the aetiology (Wang et al., 2020), we only included patients with cirrhosis caused by HCV, not patients with cirrhosis from other causes (chronic hepatitis B, alcohol intake, moderate to severe fatty liver, autoimmune liver disease or any other aetiology).

In conclusion, seroreversion of anti‐HEV IgG antibody is frequent in cirrhotic population.

HEV infection does not seem to be associated with death or liver decompensation in this population of our setting. People at high risk of developing a severe course of HEV infection or the chronification of it (such as cirrhotic) must be specifically informed of the risk involved in eating undercooked pork products and game animals, and to avoid the consumption of these products, even in those carrying anti‐HEV IgG antibodies because of the high rate of seroreversion.

## CONFLICT OF INTEREST

The authors declare no conflict of interest.

## AUTHOR CONTRIBUTIONS

ARJ had full access to all of the data in the study and takes responsibility for the integrity of the data and the accuracy of the data analysis. ARJ conceptualized and designed the study. IM, AC, IPV and AR recruited patients. PLL, MAR, JC, JCG, IGB, MF and ARJ collected samples and performed procedures. PLL, MF and ARJ analysed and interpreted the data. PLL and ARJ drafted the manuscript. All authors critically revised the manuscript for important intellectual content. PLL and ARJ performed statistical analysis. ARJ and AR obtained funding.

## Supporting information


**Supplementary Table 1**. IgG/IgM antibody levels in U/ml (units/millilitres) of the positive samples in the time line of the study.

**Supplementary Table 2**. CD4 cell levels in patients who presented seroconversion of IgM antibodies

## Data Availability

The data that support the findings of this study are available from the corresponding author upon reasonable request.
