# Supplementary material for: Seroreversion of IgG anti‐HEV in HIV cirrhotic patients: A long‐term multi‐sampling longitudinal study
Source: Transbound Emerg Dis. 2022 Mar 3;69(5):e1541–8. doi: 10.1111/tbed.14486 (PMC9790577; doi:10.1111/tbed.14486)
Supplement: Supplementary file 1 — Supplementary Table 1. IgG/IgM antibody levels in U/ml (units/millilitres) of the positive samples in the time line of the study. [file TBED-69-e1541-s002.docx]

**Supplementary Table 1.** IgG/IgM antibody levels in U/mL (units/millilitres) of the positive samples in the time line of the study.

| Patient ID | Visit 1  (IgG/IgM) | Visit 2  (IgG/IgM) | Visit 3  (IgG/IgM) | Visit 4  (IgG/IgM) | Visit 5  (IgG/IgM) | Visit 6  (IgG/IgM) | Visit 7  (IgG/IgM) | Visit 8  (IgG/IgM) | Visit 9  (IgG/IgM) | Visit 10  (IgG/IgM) | Interpretation |
| --- | --- | --- | --- | --- | --- | --- | --- | --- | --- | --- | --- |
| 1 | Neg/Neg | Neg/Neg | Neg/Neg | Neg/Neg | Neg/Neg | **54.1**/Neg |  |  |  |  | IgG seroconversion |
| 2 | Neg/Neg | Neg/Neg | Neg/Neg | Neg/Neg | Neg/Neg | **>125/70.2** |  |  |  |  | IgG and IgM seroconversion |
| 3 | **52**/Neg | **53.7**/Neg | **>125**/Neg | **51**/Neg | **51.6**/Neg | Neg/Neg | Neg/Neg |  |  |  | IgG seroreversion |
| 4 | **56.7**/Neg | **87.7**/Neg | **45.2**/Neg | Neg/Neg | Neg/Neg | Neg/Neg | Neg/Neg | Neg/Neg |  |  | IgG seroreversion |
| 5 | **52.2**/Neg | Neg/Neg | Neg/Neg | Neg/Neg | Neg/Neg | Neg/Neg | Neg/Neg | Neg/Neg |  |  | IgG seroreversion |
| 6 | **45.8**/Neg | Neg/Neg | **58.3**/Neg | Neg/Neg |  |  |  |  |  |  | IgG intermittent seroreversion |
| 7 | **124**/Neg | Neg/Neg | Neg/Neg | Neg/Neg | Neg/Neg | **101.7**/Neg |  |  |  |  | IgG intermittent seroreversion |
| 8 | **>125**/Neg | **>125**/Neg | **>125/81.5** | **41.6**/Neg | **>125/94.2** |  |  |  |  |  | IgG persistence/ IgM intermittence |
| 9 | **>125**/Neg | **>125**/Neg | **>125**/Neg | **>125**/Neg | **>125**/Neg | **>125**/Neg | **>125**/Neg | **>125**/Neg | **>125**/Neg | **>125**/Neg | IgG persistence |
| 10 | **75.7**/Neg | **65.4**/Neg | **57.5**/Neg | **82.5**/Neg | **72.9**/Neg | **57.5**/Neg | **58.3**/Neg | **55.7**/Neg | **33.7**/Neg | **60.4**/Neg | IgG persistence |
| 11 | **52**/Neg | **50.6**/Neg | **52**/Neg | **38.7**/Neg | **>125**/Neg | **46.2**/Neg | **33.7**/Neg | **45**/Neg |  |  | IgG persistence |
| 12 | **94.5**/Neg | **98.3**/Neg | **90.8**/Neg | **90.4**/Neg | **105.1**/Neg |  |  |  |  |  | IgG persistence |
| 13 | **54**/Neg | **107.5**/Neg | **80.4**/Neg | **68.2**/Neg |  |  |  |  |  |  | IgG persistence |
| 14 | **112.5**/Neg | **>125**/Neg | **>125**/Neg | **>125**/Neg |  |  |  |  |  |  | IgG persistence |
| 15 | **43.7**/Neg | **73.2**/Neg | **43.9**/Neg |  |  |  |  |  |  |  | IgG persistence |
