# Supplementary material for: Seroreversion of IgG anti‐HEV in HIV cirrhotic patients: A long‐term multi‐sampling longitudinal study
Source: Transbound Emerg Dis. 2022 Mar 3;69(5):e1541–8. doi: 10.1111/tbed.14486 (PMC9790577; doi:10.1111/tbed.14486)
Supplement: Supplementary file 2 — Supplementary Table 2. CD4 cell levels in patients who presented seroconversion of IgM antibodies [file TBED-69-e1541-s001.docx]

**Supplementary Table 2.** CD4+ cell levels in patients who presented seroconversion of IgM antibodies.

| Patient ID | Visit 1  (cells/μL) | Visit 2  (cells/μL) | Visit 3  (cells/μL) | Visit 4  (cells/μL) | Visit 5  (cells/μL) | Visit 6  (cells/μL) |
| --- | --- | --- | --- | --- | --- | --- |
| 2 | 216 | 125 | 222 | 163 | 153 | **256*** |
| 8 | 503 | 475 | **500*** | ND | **956*** |  |

***Moment when patients presented IgM anti-HEV antibody.**

No Data, ND; cells per microlitre, cells/μL
